# An empirical research on relationships between subjective judgement, technology acceptance tendency and knowledge transfer

**DOI:** 10.1371/journal.pone.0183994

**Published:** 2017-09-08

**Authors:** Yu-Hsi Yuan, Sang-Bing Tsai, Chien-Yun Dai, Hsiao-Ming Chen, Wan-Fei Chen, Chia-Huei Wu, Guodong Li, Jiangtao Wang

**Affiliations:** 1 Department of Business Administration, Yuanpei University of Medical Technology, Hsinchu, Taiwan; 2 Zhongshan Institute, University of Electronic Science and Technology of China, Guangdong, China; 3 Business and Law School, Foshan University, Guangdong, China; 4 Economics and Management College, Civil Aviation University of China, Tianjin, China; 5 Department of Industrial Education, National Taiwan Normal University, Taipei, Taiwan; 6 Institute of Service Industries and Management, MingHsin University of Science and Technology, Hsinchu, Taiwan; Southwest University, CHINA

## Abstract

The purpose of this study was to explore the relationships among employees' usage intention pertaining to mobile information devices, focusing on subjective judgement, technology acceptance tendency, information sharing behavior and information transfer. A research model was established to verify several hypotheses. The research model based on integrated concepts of knowledge management and technology acceptance modeling. Participants were employees of enterprises in Taiwan, selected by combining snowball and convenience sampling. Data obtained from 779 e-surveys. Multiple-regression analysis was employed for hypothesis verification. The results indicate that perceived ease-of-use of mobile devices was affected by computer self-efficacy and computer playfulness directly; meanwhile, perceived ease-of-use directly affects perceived usefulness. In addition, perceived ease-of-use and perceived usefulness can predict information-sharing behavior in a positive manner, and impact knowledge transfer as well. Based on the research findings, it suggested that enterprises should utilize mobile information devices to create more contact with customers and enrich their service network. In addition, it is recommended that managers use mobile devices to transmit key information to their staff and that they use these devices for problem-solving and decision-making. Further, the staff’s skills pertaining to the operation of mobile information devices and to fully implement their features are reinforced in order to inspire the users' knowledge transfer. Enhancing the playfulness of the interface is also important. In general, it is useful to promote knowledge transfer behavior within an organization by motivating members to share information and ideas via mobile information devices. In addition, a well-designed interface can facilitate employees' use of these devices.

## Introduction

When taking a comprehensive view of the knowledge economy era, it is clear that knowledge has become an important competitive element [1][2]. To knowledge intensive organizations, knowledge, even though, was the core of their key competency [3]. The American Productivity and Quality Center (APQC) defines knowledge management as a behavior that systematically enables information and knowledge to grow, flow, and further produce values [4]. The act of knowledge management is “a process to deliver right knowledge to right people at right time, and to help its sharing and further improve organizational operations” as specified by O'Dell & Hubert [5]. In the report “The Knowledge-based Economy” from The OECD (Organization for Economic Co-operation and Development) [6]: it is suggested that an economical structure based on knowledge and information acting as the foundations for production, distribution and utilization will be a main model for future economic development. Knowledge-based output is already contributing to more than 50% of the OECD member nations’ GDP (Gross Domestic Product). Economic growth and productivity enhancements in organizations rely on effective knowledge management of manpower and information technology[7].

Snowden [8]: as well as Gorelick, Milton and April [9]: note that the development of knowledge management has developed through several phases: the information supporting decision making phase, the implicit and explicit knowledge transfer phase, and lastly, knowledge development synthesis. Wong [10] points out that in a developed information and communications environment, information transfer is much faster and without frontiers. Knowledge promotes organizational knowledge more intensively and becomes the most important driver for enterprise success. The purpose of data transmission was to provide a comprehensive information for decision making via members’ analysis process. Finally, the information to be an essential mechanism for value and wisdom decision making [11–15].

In addition, the global economy has been affected by the 911 events in the United States, the Iraq and Afghanistan wars, China’s rise of economic power, global warming, the near-collapse of the global economy as well as major developments pertaining to new technology networks and broadband. In addition, factors such as the rapidly growing popularity of digital reading, more advanced mobile devices and smart phones have affected how knowledge is transferred. Although core aspects of modern knowledge management have not fundamentally been altered, there is more variation in regard to implementation approaches [5]. UK engaged in mobile technology from early 1940’s. However, until 1990’s to gain concrete prototype due to the limitation of technology development. The portable device was too big to carry out in the pocket. Dependent on the progress of information and communication technology that portable device to be implemented into commerce and decision making field as part of knowledge management [16]. O'Sullivan [17] points out that the integration of knowledge management and mobile information devices makes it possible to conduct knowledge management and knowledge sharing outside of the office setting. It is further emphasized that with mobile information devices, an organization's members can conduct knowledge management anytime and anywhere, and therefore master information sharing and knowledge transfer better, leading to more effective decision making [18–21].

By reviewed knowledge management literature [22–28]: it found that most empirical studies focus on issues related to key success factors pertaining to knowledge management in large enterprises, especially on effects of transfer and post-transfer of exclusive knowledge in organizations. There have been fewer studies related to key success factors pertaining to implementing knowledge management systems for small and medium-sized businesses. Davenport and Klahr [29]: Wong [30] point out that the success factors for implementing knowledge management in large enterprises versus small and medium enterprises are different. Due to their size, large enterprise organizations have designated departments or personnel who are required to promote knowledge management activities. Due to limited resources, small and medium-sized enterprise organizations need more specific decisions take in regard to implementation; therefore, the leader is of key importance when it comes to promoting knowledge management. David & Chesebrough [31] note that knowledge management can enhance the competitive advantage for small and medium-sized enterprises. In an empirical study, Edvardsson [32] found that for small and medium-sized enterprises, knowledge management can not only improve decision quality, enhance productivity, raise market share and lower cost, but also enhance profit and innovation. Obviously, Knowledge sharing is the priority in the knowledge management [33]. Yet, Knowledge sharing behavior to be impacted by working environment positively [34]. In practical, the goal for enterprises to promote knowledge was focus on organization change, process improvement, enhance the quality of product or service, or cultivating their competencies [35–36]. Because of the serious situation of changing market, globalization and competiveness, employees’ turnover rate etc., therefore, knowledge gain, knowledge transfer, and knowledge creation become the priority for enterprise to coping with such issues positively [37–40].

Compare with the small and medium enterprises (SMEs) in Taiwan, SMEs of other countries were engaged in knowledge management conceptual construction for cultivating their competency [41–42]. A dramatic development for SMEs in Taiwan dependents on the input of resource from Taiwan Government, however, the performance and outcome need to further evaluation [43]. In a review of Taiwan's economic development survey in “White Paper of Small and Medium Enterprises” [44] indicate that in 2013, Taiwan's SME business and employment shows growth positively. Report reflect that SMEs reached a new peak of 1,330,000, which accounted for 97.64%, and total 8,588,000 to be haired with employment rate of 78.3%. This shows that small and medium-sized enterprises are playing a major role in the economic growth in Taiwan. In recent years, European and US markets have been declining, often affecting the profitability of overseas orders for Taiwanese enterprises. Therefore, small and medium-sized enterprises have to expand actively into overseas markets. Through knowledge transfer, optimal efficacy for enterprises can obtained. This will maintain operational profitability. Therefore, issues related to implementing knowledge management in small and medium-sized enterprises become more and more important. Thus, this paper primarily focuses on knowledge management, specifically discussing the use of mobile information devices as media for knowledge transfer, and explores cause-effect relationships among the following variables pertaining to current employees of Taiwanese enterprises: “subjective judgement” and “technology acceptance tendency”, “information sharing behavior” and “knowledge transfer”. The goal is to promote effective models for organizational communication and decision-making quality and speed. The study results expected to provide material for further use in academic studies and to enhance enterprise knowledge management decision pertaining to related applications.

## Theoretical background and hypothesis

In order to enhance their competitive advantage, organizations systematically preserve and apply their own knowledge through information systems. This is also an important approach for enterprises when it comes to implementing knowledge management [29][45]. Even if an organization vigorously promotes knowledge management in order to use accumulated knowledge to enhance their competitiveness, the organizational members' willingness to embrace such knowledge transfer is an important factor for the organization in promoting knowledge management [46][47]. In order to evaluate the tendency of organizational members to use technology for conducting knowledge management, a technology acceptance model is used. The behavioral intention of an organization's members pertaining to conducting knowledge transfer becomes an important measure; hence, the related literature to be discussed below.

### The relationship among external variables and the technology acceptancemodel

In order to effectively explain and predict the behavioral intention of information technology users, Davis [48] applied Fishbein and Ajzen’s [49] rational behavior theory and planned behavior theory as a basis, combined with the Technology Acceptance Model (TAM) applied to information systems.

With TAM as a starting point, Venkatesh and Davis [50] developed the TAM2 model. They included societal influences (personal standards, autonomy, image) and perception tool flows (subjective judgement, output quality, result clarity and perceived usefulness): as well as behavioral model-related theories (action theory, work motivation theory, behavior decision theory) in order to define and develop cause and effect relationship models. However, action theory, work motivation theory and behavior decision theory all show that behaviors connected from mental representation to tools; hence, the extended specific behavior model will be linked to higher level personal work objectives. Based on TAM2, the model will use mental representations in order to evaluate acts between important work objectives and use of the system, showing a pattern of basic behavior for responsive judgment to use performance (i.e. “perceived usefulness”). Hence, the study considers that “subjective judgement” combined with perceived judgment. In other words, a direct effect applied on “perceived usefulness”, means that in their experience, user acceptance has already been connected to “subjective judgement”, where it includes the importance to work persistent [51]: mission skill fitness [52]: and perceived fitness [53]. In addition, Kim’s [54] empirical study on the relationship between mobile phone use and work included studying aspects covering various business sectors. The results show that no matter what kind of profession, “subjective judgement” viewed as an important factor in considering technology's practicality. Davis [48] also shows that whether people tend to use or not use the application depends on whether it will be helpful to job execution. Apparently “perceived usefulness” can be treated as “the degree to which a person believes that using a specific system will enhance his or her work”. Hence, the study raises the following hypotheses:

H1 null hypothesis: The “subjective judgement” has a significant positive effect on “perceived usefulness”.
H1 alternative hypothesis: The “subjective judgement” no positive effect on “perceived usefulness”.

Further, Bandura [55] discusses “self-efficacy”, which is considered be an individual's capability judgment toward achieving specific work, indicating that such capability will change along with individual growth and experiences. Wangpipatwong, Chutimaskul and Papasratorn [56] propose “computer self-efficacy” as a significant factor for an individual determining to use a computer. Compeau and Higgins [57] consider “computer self-efficacy” to have an important role in forming personal feelings and behaviors. Venkatesh [58] considers “computer self-efficacy” as an important decision factor pertaining to “perceived ease-of-use”. In the literature on user technology acceptance, the most frequently explored relationship is that of the effect of “computer self-efficacy” on “perceived ease-of-use” (e.g. Venkatesh & Davis [50]; Venkatesh [58]). This literature also focuses a lot on “perceived ease-of-use”. Yi and Hwang [59]: Summarizing previous studies, found that technology acceptance behavior studies mainly focused on the effects of “self-efficacy” on “perceived ease-of-use” [50]: and lack of self-evaluation [57–58]. This indicates that individual high computer self-efficacy will raise an individual’s computer use frequency, using it for more recreational activities and experience less computer anxiety. Hence, this study proposes the following hypothesis:

H2 null hypothesis: The “computer self-efficacy” has a significant positive effect on “perceived ease-of-use”.
H2 alternative hypothesis: The “computer self-efficacy” has no positive effect on “perceived ease-of-use”.

It was further noted in related literature that “computer playfulness” influence use behavior [60–63]. In the research model of Yi and Hwang [59]: computer playfulness and computer anxiety were not originally included, while both two variables found to have the effect of dominating system use experience toward “perceived ease-of-use” [64]. Therefore, Venkatesh [58] proposed to bring “computer playfulness” in as a decisive factor in “perceived ease-of-use”. Davis et al. [65] found that “computer playfulness” significantly affects computer technology acceptance. When studying the difference between gaming and traditional training, Venkatesh [66]: found that a higher level of “perceived ease-of-use” existed in game training. Because game training is able to induce the user with higher level of interest and thus induces internal motivation in favor of generating ease of use perception, Venkatesh [50] proposed that internal motivation involves the user's cognitive “perceived ease-of-use”. When synthesizing related study results, it found that system use experiences affect an individual’s interest in using the system and significantly affect personal perception of system complexity, showing that interest guides a system user toward a “perceived ease-of-use” experience [64]. Related studies support the relationship between user internal motivation and computer use efficacy through “computer playfulness” [67]. Furthermore, in related literature, the effect of “computer playfulness” on “perceived ease-of-use” had verified, for example in Igbaria, Parasuraman and Baroudi [68]. They found that the user tends to utilizes “computer playfulness” as intrinsic motivation for computer use. Their results indicate that “computer playfulness” and use behavior have a positive correlation. Moon and Kim [69] extends the technology acceptance model and applied it to the World-wide Web, and found that in addition to “perceived ease-of-use” and “perceived usefulness”, “computer playfulness” is a factor affecting one's attitude towards the Internet. It found that three factors all have a positive effect on user computer attitude, and that “computer playfulness” was further affected by “perceived ease-of-use” directly. Accordingly, the study raises the following hypothesis:

H3 null hypothesis: The “computer playfulness” has a significant positive effect on “perceived ease-of-use”.
H3 alternative hypothesis: The “computer playfulness” has no positive effect on “perceived ease-of-use”.

### The impact of the technology acceptance model on user behavior tendency and information sharing

TAM mainly used to explain, evaluate and predict a user's acceptance of information system [48–49]. Davis [48] adopts original rational behavior theory and planned behavior theory as basis for his model and re-inspecting the user’s computer acceptance theory, thus proposing a technology acceptance model modification, proposing that “perceived usefulness” and “perceived ease-of-use” are important factors influencing user technology acceptance. Davis [48] develops a proper rating scale for the two variables “perceived usefulness” and “perceived ease-of-use”, and conducted related empirical research. These arguments supported in analysis results of two studies by Adams, Nelson and Todd [70]. Specifically, when using rating scales, study results support that psychological properties of “perceived usefulness” and “perceived ease-of-use” confirm the assumptions, and that a cause-effect relationship exists between these two variables. Several empirical studies have adopted TAM as their theory basis, including Adamson and Shine [71] who explore banking finance agents' user satisfaction with terminal information systems. Ahn, Ryu and Han [72] studied user behavior of web shopping; Brown and Jayakody [73] explores e-commerce user behavior toward B2C (Business to Customer) information systems. All results verify that “perceived ease-of-use” have a significant positive effect on “perceived usefulness”. Accordingly, the study proposes the following hypothesis:

H4 null hypothesis: The “perceived ease-of-use” will affect “perceived usefulness” positively.
H4 alternative hypothesis: The “perceived ease-of-use” will not affect “perceived usefulness” positively.

Davis’ [48] found that the correlation between “perceived usefulness” and user behavior is significantly higher than that of “perceived ease-of-use” and user behavior. Venkatesh and Davis [74] point out that in TAM assumptions “use behavior intension” affected by “perceived ease-of-use”. In addition, user’s cognition needs toward endeavor level and “perceived usefulness” and, to some extent, use of the system, enhance the user’s personal cognition and self-work performance.

Venkatesh and Davis [74] propose that TAM is an adaptive adjustment of Theory of Reasoned Action (TRA): in that the objective is to understand external variables related to the user’s information technology acceptance and actual system use behavior at the work site. External variables such as subjective judgement, computer self-efficacy, computer playfulness, user participation in system design and development. To generate an effect at a certain level toward “perceived usefulness” and “perceived ease-of-use” [49][75]. Jackson, Chow and Leitch [76] point out that in the TAM model “use behavior intention” influences the attitude of the system user and their subjective cognition, proving with predictive power that this variable enhances personal work performance.

In a study from 2008 evaluating user acceptance of advanced mobile communication services, López-Nicolás, Molina-Castillo and Bouwman [77] find that information technology acceptance frequently affected by “use behavior intention”. Also, a study of continuous use of mobile networks [78]: a study of use of online learning by hi-tech company engineers [79]: and a study of consumers' attitudes toward using mobile TV service [80] all found that both “perceived ease-of-use” and “perceived usefulness” have a positive effect on “use behavior intention”. Thus, the study proposes the following hypotheses:

H5 null hypothesis: The “perceived usefulness” has a significant positive effect on “use behavior intention”.
H5 alternative hypothesis: The “perceived usefulness” has no positive effect on “use behavior intention”.
H6 null hypothesis: The “perceived ease-of-use” has a significant positive effect on “use behavior intention”.
H6 alternative hypothesis: The “perceived ease-of-use” has no positive effect on “use behavior intention”.

Erdelez and Rioux [81] define “information sharing” as a complete process of an individual sharing his/her own information, or acquired information from others. Besides, Erdelez and Rioux [81] argued that information sharing and gaining should combined as a sound information sharing behavior. Both behaviors must combine in order to have complete information sharing behavior [82]. Davis and Venkatesh [83] Venkatesh and Davis [50] propose that when “perceived usefulness” focused on knowledge transfer in businesses or professional fields, this pertains to small range and centralized focus-type of information and represents cognition at a higher level. The meaning of “perceived ease-of-use” pertains more to personal leisure and everyday life information sharing, focusing on large range and popular information and representing lower-level cognition. In the knowledge economy era of keen competition, enterprise organizations must effectively acquire, store, accumulate, share and apply knowledge, to ensure operational advantages [84]. Currently, networks are developing quickly. Therefore, at the individual level, information sharing behavior needs to exert more efficacy in order to influence society and organizations in a powerful manner. Thus, the study proposes the following hypothesis:

H7 null hypothesis: The “perceived ease-of-use” variable has a significant positive effect on “information sharing behavior”.
H7 alternative hypothesis: The “perceived ease-of-use” variable has no positive effect on “information sharing behavior”.

### The effectiveness of information sharing behavior on knowledge transfer

Quinn, Anderson and Finkelstein [85] consider that in the post-industrial era, organizational key success factors have been transferred from tangible assets management to human intelligence and systems management because growth of new economic industries are mostly created by the expertise of specialized professionals. Nonaka and Konno [86] define “knowledge transfer” as interaction of implicit and explicit knowledge among individuals and organizations, and thus generating effective knowledge transfer. Gilbert and Gordey-Hayes [87] consider that “knowledge transfer” must go through a dynamic learning process involving knowledge acquisition, communicating, acceptance and assimilation phases in order to function properly. In Kramer and Wells' [88] empirical study, it found that “knowledge transfer” develops via participative ergonomics with network relationships among members. In the transfer process, participative ergonomics can use to observe and record the “knowledge transfer” process, and then combine this with networks so that specific procedures and steps can be constructed for the transfer process. Hsiao, Tsai, and Lee [89] consider that “knowledge transfer” is an expert practice pertaining to work content that through certain transmission methods leads to complete knowledge transfer, achieving the objective of the knowledge transmission. Davis and Venkatesh [83] note that “information sharing” mainly means sharing behavior pertaining to low-level actions. While “knowledge transfer” content is more focused on practicality, i.e., the so-called “perceived usefulness”; if compared to “information sharing”, “knowledge transfer” is sharing specific to a certain theme. Venkatesh and Bala [90] propose that “knowledge transfer” pertains to high-level goals. Newell [91], Musen [92], and Senge [93] adopted the use of “knowledge base system perspective” and “learning perspective” pertaining to various perspectives in order to explain knowledge transfer. Based on the perspectives presented above, this paper considers “information sharing behavior” focused on computer knowledge-based sharing and repeated use. Focusing on computers equipped with a standard data format, there is no need to consider the problem of whether receiver is able to receive or not. While Senge’s [93] “learning perspective” focused on empiricism and considering that knowledge cannot be independent from the context, it is here considered that knowledge transfer must take place in interaction with the opposite side or group, so that after successful transfer, it will manifest as action capability of the other party. Accordingly, the study proposes the following hypotheses:

H8 null hypothesis: The “use behavior intention” variable has a significant positive effect on “information sharing behavior”.
H8 alternative hypothesis: The “use behavior intention” variable has no positive effect on “information sharing behavior”.
H9 null hypothesis: The “information sharing behavior” variable has a significant positive effect on “knowledge transfer”.
H9 alternative hypothesis: The “information sharing behavior” variable has no positive effect on “knowledge transfer”.

## Research design

### Instrument development

The instrument consisted of “perceived usefulness” (4 items): “perceived ease-of-use” (3 items): “subjective judgement” (4 items): “computer self-efficacy” (4 items): and “computer playfulness” (4 items) which were retrieved from Venkatesh and Bala (2008). Moreover, “usage intention” (4 items) was obtained from López-Nicolás, Molina-Castillo and Bouwman [77]; “information sharing behavior” (3 items) was gained from Yu, Lu and Liu [94]: and “knowledge transfer” (3 items): was selected from Lee, Lee and Kang [95]. Hence, the instrument contained 8 factors and 30 items in total. The Likert 5-point scale was employed as the ranking system from “strongly agree” (5 points) to “strongly disagree” (1 point). The expertise committee invited four professors, two national level industry researchers, one enterprise general manager, two enterprise officers and three educational administrators to evaluate the content validity. The Cronbach’s α for the instrument was 0.934. The sub-scales were 0.760 to 0.953 and all passed the validity criteria [96–98].

### Instrument validation

Regarding to instrument validation, confirmatory factor analysis (CFA) used in verification of convergent validity and discriminant validity. Anderson and Gerbing [99] suggest that an instrument that passes the CFA based one-factor loading test reaches the significant level (*p* < .05) via the model’s convergent validity verification. The results show that all items reached the significant level, reflecting that the items belonging to each factor are valid in measuring the same concept (see Table 1). The composite reliability (CR) and average variance extracted (AVE) used to test the validity and reliability of the instrument as well [100]. According to Bagozzi and Yi’s [101] recommendations, the CR and AVE values should be greater than .50 in order to demonstrate that the item quality is acceptable. The test results show that all but one factor passed the standard (see Table 1); only the CR value for “computer playfulness” was slightly lower than required. However, in order to retain the integrity of the instrument, this this factor was included.

**Table 1:** Summary of convergent validity verification coefficients.

Factors	Coefficient	Factor loading	ρ_c_ (composite reliability)	AVE (average variance extracted)
Subjective judgement (SJ)	λ_1_	0.825***	.728	.402
	λ_2_	0.789***		
	λ_3_	0.769***		
	λ_4_	0.798***		
Computer self-efficacy (CSE)	λ_1_	0.699***	.574	.255
	λ_2_	0.635***		
	λ_3_	0.737***		
	λ_4_	0.754***		
Computer playfulness (CPLAY)	λ_1_	0.809***	.435	.188
	λ_2_	0.512***		
	λ_3_	0.677***		
	λ_4_	0.457***		
Perceived ease-of-use (PEOU)	λ_1_	0.808***	.590	.336
	λ_2_	0.615***		
	λ_3_	0.814***		
Perceived usefulness (PU)	λ_1_	0.868***	.878	.583
	λ_2_	0.910***		
	λ_3_	0.863***		
	λ_4_	0.851***		
Usage intention (UI)	λ_1_	0.847***	.601	.310
	λ_2_	0.863***		
	λ_3_	0.566***		
	λ_4_	0.509***		
Information sharing behavior (ISB)	λ_1_	0.770***	.738	.487
	λ_2_	0.873***		
	λ_3_	0.852***		
Knowledge transfer (KT)	λ_1_	0.867***	.896	.683
	λ_2_	0.879***		
	λ_3_	0.932***		
	λ_4_	0.950***		

Anderson and Gerbing [99] note that pairwise factor comparison used in order to determine discriminate validity. Constrain the correlation of pairwise factors as 1.0 (constrained model): then estimate the correlation of pairwise factors (free estimated model). Finally, theχ^2^ discrepancy coefficients (Δχ^2^) should be verified through the constrained model and free estimated model. When the free estimated model give lower values than the constrained model, it demonstrated that the instrument passed the discriminate validity test. Further, Anderson and Gerbing [99] argue that a 95% confidence interval (CI) should checked; if the CI value does not exceed 1.0, discriminate validity had demonstrated.

The significance level (.05) divided by test times was used to obtain a coefficient of .005 (.05/10) as the new criteria for the Bonferroni correction procedure. This used in order to avoid the type I error risk increase when the hypothesis to be tested repeatedly [102]. The results show that the 8 factors/ 36 times comparison coefficient Δχ^2^ is from 48.590 to 608.694, and that Δdf = 1 (freedom degree discrepancy) reached the significant level (*p* < .05). Meanwhile, none of the CI of 8 factors crossed 1.0 (see Table 2): which supported the discriminate validity of the instrument.

**Table 2:** Summary of discriminant validity verification coefficients.

Pairwise factors		Constrained model		Free estimated model		Δ*χ*^2^	Confidence interval (CI)	
		*χ*^2^	*df*	*χ*^2^	*df*		Lower level	Upper level
SJ	JR	441.312***	14	155.999***	13	285.313	.519	.674
	CSE	618.591***	20	111.356***	19	507.235	.148	.312
	CPLAY	811.091***	20	202.397***	19	608.694	.160	.327
	PEOU	569.737***	14	91.987***	13	477.750	.104	.271
	PU	483.536***	20	171.998***	19	311.538	.333	.508
	UI	648.443***	20	228.394***	19	420.049	.123	.301
	ISB	550.840***	14	115.295***	13	435.545	.271	.438
	KT	392.348***	20	185.395***	19	206.953	.547	.680
CSE	CPLAY	544.587***	20	381.270***	19	163.317	.434	.725
	PEOU	251.222***	14	43.620***	13	207.602	.504	.674
	PU	555.218***	20	60.526***	19	494.692	.263	.440
	UI	573.349***	20	138.697***	19	434.652	.420	.580
	ISB	202.053***	14	63.264***	13	138.789	.400	.559
	KT	643.245***	20	130.711***	19	512.534	.158	.319
CPLAY	PEOU	351.902***	14	232.796***	13	119.106	.373	.586
	PU	236.195***	20	182.409***	17	53.786	.205	.382
	UI	484.519***	20	298.188***	19	186.331	.451	.627
	ISB	312.672***	14	188.094***	13	124.578	.356	.532
	KT	290.367***	20	232.785***	19	57.582	.194	.384
PEOU	PU	77.825***	14	29.235***	13	48.590	.201	.390
	UI	565.295***	14	118.837***	13	446.458	.257	.426
	ISB	71.515***	9	7.878***	8	63.637	.238	.401
	KT	592.975***	14	106.908***	13	486.067	.100	.271
PU	UI	628.969***	20	177.001***	19	451.968	.120	.301
	ISB	159.624***	14	37.483***	13	122.141	.329	.495
	KT	440.039***	20	131.881***	19	308.158	.358	.504
UI	ISB	640.668***	14	203.092***	13	437.576	.355	.533
	KT	668.338***	20	290.579***	19	377.759	.213	.385
ISB	KT	530.189***	14	115.653***	13	414.536	.330	.480

SJ = Subjective judgment; CSE = Computer self-efficacy; CPLAY = Computer playfulness; PEOU = Perceived ease of use; PU = Perceived usefulness; UI = Usage intention; ISB = Information sharing behavior; KT = Knowledge transfer.

### Sampling

The employees who invited to participate in this study selected from small and medium enterprises (SME) in Taiwan. Because it was difficult to require enterprise workers to participate and the return rate was relatively low [103]: snowball and convenience-sampling methods combined to collect data. The e-survey's web address was forward to the researchers' colleagues and employees at other companies, asking ask them to participate in this study. Data collection took place from July 20 to September 20, 2012. Total 1,100 forms returned, from which 779 valid questionnaires selected for data analysis. The rest of the questionnaires were of low quality, including missing answers or inconsistencies among answered items.

### Hypothesis model

**Fig 1:**
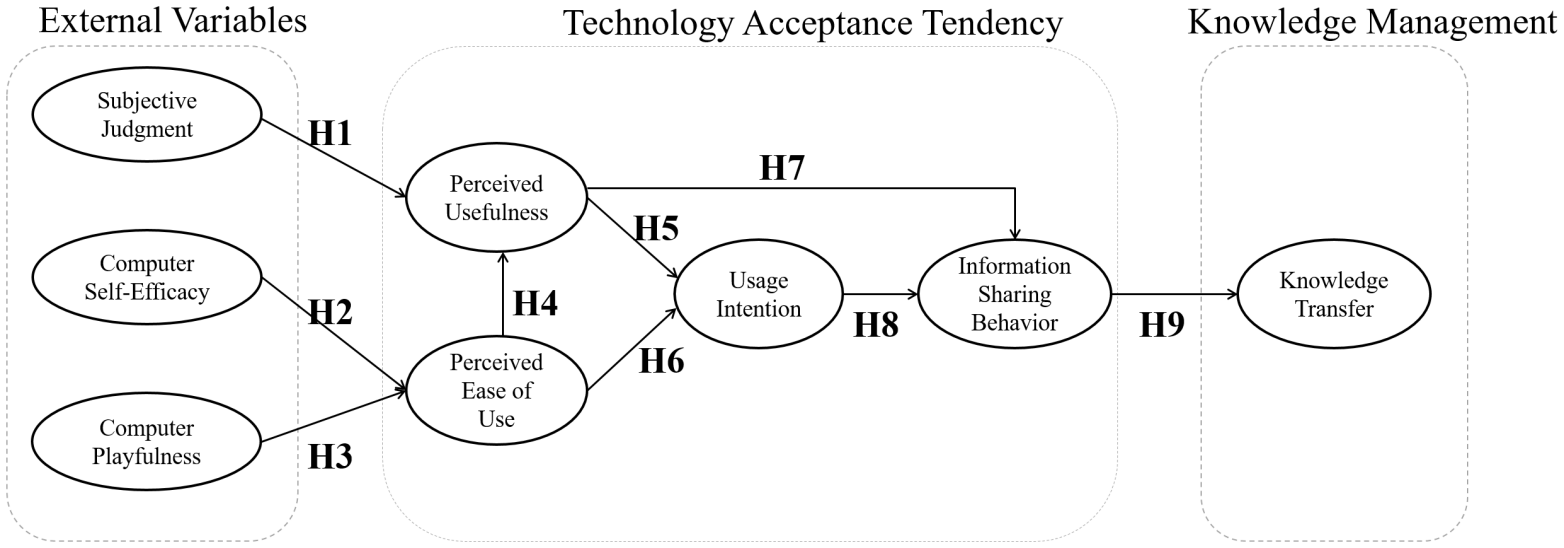
Hypothesis model.

The proposed model (Fig 1) established based on theory. The technology acceptance model is the core theory used in this study. In addition, various external variables explored, including “subjective judgement”, “computer self-efficacy” and “computer playfulness”. These affected internal variables of technology acceptance tendency at the individual level, such as “perceived usefulness”, “perceived ease-of-use”, “usage intention” and “information sharing behavior”. Then, the dependent variable, “knowledge transfer”, predicted at the group level based on the individual level variable “information sharing behavior”.

## Results

### Sample background

Regarding to characteristics of demographic variables (see Table 3): the number of males and females was about equal (male = 50.8%, female = 49.2%). Employees between the ages of 25 and 44 (75.4%) made up the main age group. Employees who had earned a bachelor's degree (49.6%) made up the largest group in regard to education. Employees in the educational service field (38.9%) were in the majority. The prevalent company size was having 201 or more employees (58.2%). The largest group when it came to seniority was those with less than 3 years' experience (39.8%). The prevalent group in this study pertaining to job responsibility consisted of front-line workers (49.9%).

**Table 3:** Profile of participants (N = 779).

Variables	Groups	n	%	Mean score of the survey			
				Mean	SD	F/t value	Post-Hoc
Gender	❶Female	383	49.2	3.79	.419	-3.892***	❷>❶
	❷Male	396	50.8	3.91	.460		
Age	❶16~24 years old	56	7.2	3.76	.418	1.059 n.s.	—
	❷25~44 years old	587	75.4	3.86	.446		
	❸45~54 years old	109	14.0	3.88	.456		
	❹Over 55 years old	27	3.5	3.80	.412		
Education	❶Senior high school or less	57	7.3	3.77	.453	.726 n.s.	—
	❷Some college	124	15.9	3.84	.452		
	❸Graduated from college	386	49.6	3.87	.442		
	❹Master	179	23.0	3.84	.447		
	❺Doctor	33	4.2	3.90	.424		
Industry	❶Educational service field	303	38.9	3.91	.457	5.305***	❶>❸ ❷>❸
	❷Financial and insurance	154	19.8	3.89	.368		
	❸Manufacturing	72	9.2	3.69	.437		
	❹Information and communication	62	8.0	3.75	.421		
	❺Other	188	24.1	3.82	.471		
Size of organization	❶Less than 5 employees	42	5.4	3.88	.466	6.520***	—
	❷6–100	185	23.7	3.75	.436		
	❸101–200	99	12.7	3.79	.427		
	❹Over 201	453	58.2	3.91	.442		
Seniority	❶Less than 3 years	310	39.8	3.85	.436	.638 n.s.	—
	❷4–6 years	166	21.3	3.89	.480		
	❸7–9 years	118	15.1	3.84	.411		
	❹More than 10 years	185	23.7	3.83	.445		
Position	❶Front-line worker	389	49.9	3.84	.451	.617 n.s.	—
	❷Technician	112	14.4	3.81	.457		
	❸Middle manager	173	22.2	3.90	.408		
	❹Senior manager	72	9.2	3.87	.498		
	❺Staff	16	2.1	3.84	.444		
	❻Other	17	2.2	3.82	.445		

n.s. p>.05;

*** p < .001

### Differential analysis

The results of differential analysis presented in Table 3. Independent t-test and One-way ANOVA conducted to examine the significant difference of the total mean score of survey. It found that male’s (M = 3.91, SD = 4.60) total mean score (t = -3.892, *p* < .001) higher than female (M = 3.79, SD = 4.19). Results reflected that male’s information usage at work tendency higher than female. In addition, the industry (F = 5.305, *p* < .001) was educational service field (M = 3.91, SD = 4.57) and financial & insurance (M = 3.89, SD = 3.68) higher than Manufacturing (M = 3.69, SD = 4.37): which reached the significant level and verified by Scheffe post-hoc. The rest characteristics did not reach the statistical significant level. Even the size of organization shows F value reached the significant level, however, Scheffe post-hoc does not support the group difference.

### Model verification

The path analysis had conducted through multiple linear regression in order to determine the correlation of pairwise factors and to test the causal relationships of the hypothesis model. The following describes the test results.

#### (1) The effect of “subjective judgement” and “perceived ease-of-use” on “perceived usefulness”

The “subjective judgement” and “perceived ease-of-use” variables had used as independent variables, and “perceived usefulness” was the dependent variable in order to determine the causal relationship (see Table 4). The result shows that the multiple regression coefficient R is .720. R^2^ is .518, which reflects that “perceived usefulness” to be predicted positively by “subjective judgement” and “perceived ease-of-use”. The explained variance reached 51.8%. The path from “subjective judgement” (*β* = .668, *p* < .001): and “perceived ease-of-use” (*β* = .095, *p* < .001) to “perceived usefulness” both reached significant levels. Moreover, the “subjective judgement” scored higher than “perceived ease-of-use” as demonstrated by standard path coefficients. The H1 and H4 null hypothesis had supported by statistical test and rejected the alternative hypotheses.

**Table 4:** Path analysis coefficient summary of “subjective judgment” and “perceived ease of use” to “perceived usefulness”.

Independent variables	Unstandardized coefficients		Standardized coefficient (*β*)	t value
	B	Standard error		
(intercept)	.929	.145		6.402***
Perceived ease of use	.126	.035	.095	3.589***
Subjective judgment	.643	.029	.668	21.971***
R = .720; R2 = .518; Adjusted R2 = .516; F = 277.763***				

*** p < .001

#### (2) The effect of “computer self-efficacy” and “computer playfulness” on “perceived ease-of-use”

The “computer self-efficacy” and “computer playfulness” variables be put as independent variables, and “perceived ease-of-use” was put as the dependent variable in order to determine the causal relationship (see Table 5). The results show that the multiple regression coefficient R is .519, and that R^2^ is .269, reflecting that “perceived ease-of-use” to be predicted positively by “computer self-efficacy” and “computer playfulness”. Further, the explained variance reached 26.9%. The path from “computer self-efficacy” (*β* = .327, *p* < .001) and “computer playfulness” (*β* = .273, *p* < .001) to “perceived ease-of-use” both reached significant levels. Moreover, the “computer self-efficacy” scored slightly higher than “computer playfulness” as demonstrated by standard path coefficients. The H2 and H3 null hypothesis had supported by statistical test and rejected the alternative hypotheses.

**Table 5:** Path analysis coefficient summary of “computer self-efficacy” and “computer playfulness” to “perceived ease of use”.

Independent variables	Unstandardized coefficients		Standardized coefficient (*β*)	t value
	B	Standard error		
(intercept)	1.779	.136		13.119***
Computer self-efficacy	.314	.034	.327	9.290***
Computer playfulness	.252	.033	.273	7.739***
R = .519; R2 = .269; Adjusted R2 = .267; F = 142.946***				

*** *p* < .001

#### (3) The effect of “perceived usefulness” and “perceived ease-of-use” to “usage intention”

The “perceived ease-of-use” and “perceived usefulness” be put as independent variables, and “usage intention” was put as the dependent variable in order to determine the causal relationship (see Table 6). The results show that the multiple regression coefficient R is .354, and that R^2^ is .125. This reflects “usage intention” could predicted positively by “perceived ease-of-use” and “perceived usefulness”. The explained variance reached 12.3%. The path from “perceived ease-of-use” (*β* = .257, *p* < .001): and “perceived usefulness” (*β* = .177, *p* < .001) to “usage intention” both reached significant levels. Moreover, the “perceived ease-of-use” scored slightly higher than “perceived usefulness”, as demonstrated by standard path coefficients. The H5 and H6 null hypothesis had supported by statistical test and rejected the alternative hypotheses.

**Table 6:** Path analysis coefficient summary of “perceived ease of use” and “perceived usefulness” to “usage intention”.

Independent variables	Unstandardized coefficients		Standardized coefficient (*β*)	t value
	B	Standard error		
(intercept)	2.105	.172		12.241***
Perceived usefulness	.153	.031	.177	5.006***
Perceived ease of use	.296	.041	.257	7.285***
R = .354; R2 = .125; Adjusted R2 = .123; F = 55.663***				

*** *p* < .001

#### (4) The effect of “perceived usefulness” and “usage intention” on “information sharing behavior”

The “perceived usefulness” and “usage intention” be put as independent variables, and “information sharing behavior” was put as the dependent variable in order to determine the causal relationship (see Table 7). The results show that the multiple regression coefficient R is .562, and that R^2^ is .315. This reflects “information sharing behavior” could predicted positively by “perceived usefulness” and “usage intention”. The explained variance reached 31.5%. The path from “usage intention” (*β* = .375, *p* < .001): and “perceived usefulness” (*β* = .333, *p* < .001) to “information sharing behavior” reached significant levels. Moreover, the “usage intention” scored slightly higher than “perceived usefulness” as demonstrated by standard path coefficients. The H7 and H8 null hypothesis had supported by statistical test and rejected the alternative hypotheses.

**Table 7:** Path analysis coefficient summary of “perceived usefulness” and “usage intention” to “information sharing behavior”.

Independent variables	Unstandardized coefficients		Standardized coefficient (*β*)	t value
	B	Standard error		
(intercept)	1.387	.131		10.559***
Perceived usefulness	.275	.025	.333	10.829***
Usage intention	.357	.029	.375	12.210***
R = .562; R2 = .315; Adjusted R2 = .314; F = 178.793***				

*** *p* < .001

#### (5) The effect of “information sharing behavior” on “knowledge transfer”

The “information sharing behavior” put as the independent variable, and “knowledge transfer” put as the dependent variable in order to determine the causal relationship (see Table 8). The results show that the multiple regression coefficient R is .452, and that that R^2^ is .204. This reflects that “knowledge transfer” could be predicted positively by “information sharing behavior” (*β* = .452, *p* < .001). The explained variance reached 20.4%. The H9 null hypothesis had supported by statistical test and rejected the alternative hypothesis.

**Table 8:** Path analysis coefficient summary of “information sharing behavior” to “knowledge transfer”.

Independent variables	Unstandardized coefficients		Standardized coefficient (*β*)	t value
	B	Standard error		
(intercept)	1.337	.167		7.999***
Information sharing behavior	.606	.043	.452	14.108***
R = .452; R2 = .204; Adjusted R2 = .203; F = 199.048***				

*** *p* < .001

**Fig 2:**
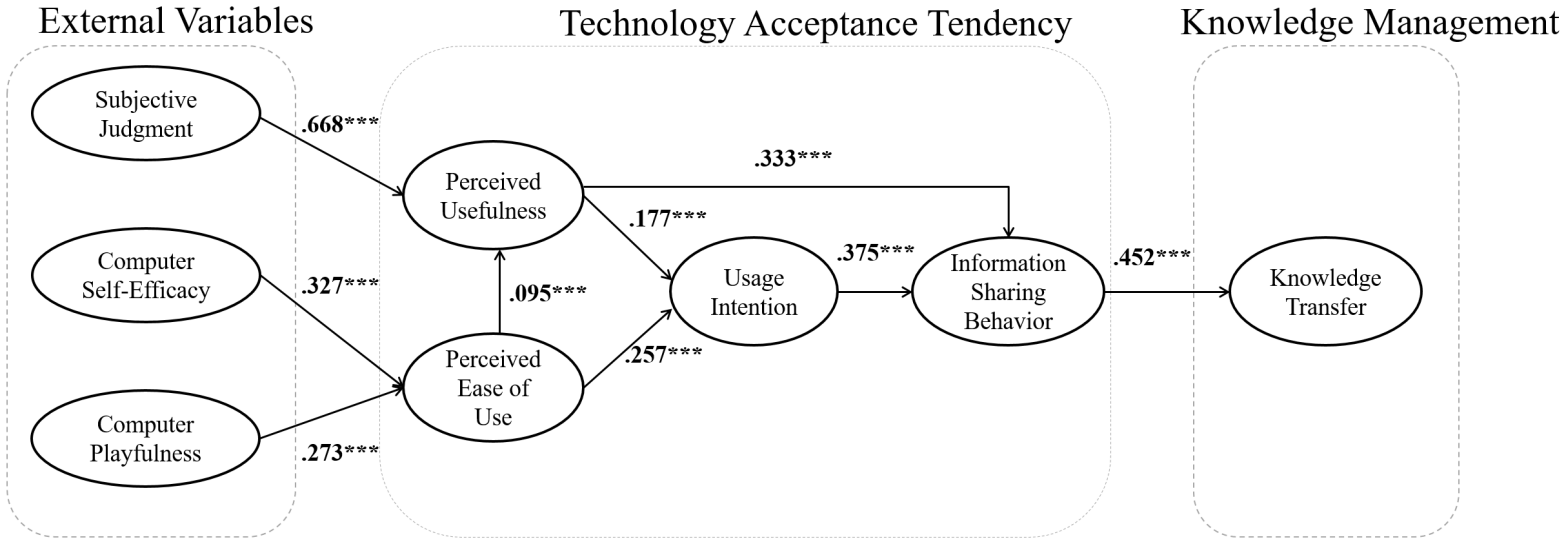
Path analysis results based on all valid samples (n = 779).

The overall model estimation results presented in Fig 2 by structural equation modeling (SEM) and the hypotheses test result presented in Table 9. All hypotheses be supported based on statistical tests. As described above, “perceived usefulness” can be predicted by “subjective judgement” (*β* = .668, *p* < .001) and “perceived ease-of-use” (*β* = .213, *p* < .001); further, “perceived ease-of-use” to be predicted by “computer self-efficacy” (*β* = .327, *p* < .001) and “computer playfulness” (*β* = .273, *p* < .001). The variable “usage intention” can be affected by “perceived usefulness” (*β* = .177, *p* < .001) and “perceived ease-of-use” (*β* = .257, *p* < .001). The variable “information sharing behavior” can be predicted by “perceived usefulness” (*β* = .333, *p* < .001) and “usage intention” (*β* = .375, *p* < .001). In addition, “knowledge transfer” will be affected by “information sharing behavior” (*β* = .452, *p* < .001). The findings demonstrate that technology acceptance tendency can facilitate organizational knowledge management according to statistical results.

**Table 9:** Hypotheses test result summary.

Hypotheses	Paths			Path coefficients	Results
H1	Subjective Judgment	→	Perceived usefulness	.668***	Supported
H2	Computer self-efficacy	→	Perceived ease of use	.327***	Supported
H3	Computer playfulness	→	Perceived ease of use	.273***	Supported
H4	Perceived ease of use	→	Perceived usefulness	.095***	Supported
H5	Perceived usefulness	→	Usage intention	.177***	Supported
H6	Perceived usefulness	→	Usage intention	.257***	Supported
H7	Perceived usefulness	→	Information sharing behavior	.333***	Supported
H8	Usage intention	→	Information sharing behavior	.375***	Supported
H9	Information sharing behavior	→	Knowledge transfer	.452***	Supported

*** *p*<0.01

### Model fit test

The goodness of fit of the proposed model (Fig 1) was satisfactory (χ^2^ = 7.762, df = 3, *p*>.05). Further, all other model fit indices were acceptable: χ^2^/df = 2.58, GFI = 0.998, AGFI = 0.970, SRMR = 0.022, RMSEA = 0.045, CFI = 0.997, NFI = 0.996, NNFI = 0.976, IFI = 0.997, Akaike’s information criterion (AIC) = 89.762, Bayesian information criterion (BIC) = 227.477, and expected cross-validation index (ECVI) = .115. The results indicated that all paths reached the statistical significant level and the perimeters equal to regression coefficients. In summary, the model test supported a good fit results.

## Conclusions and suggestions

### Academic contributions

Regarding to academic contributions, these testing results of convergent validity and discriminant validity in the scale chart show that the measurement standards had matched. With further cross-validation, similarly support in the measuring model can handle different sample groups and can be used as an academically related study tool.

Further, in terms of practice, when employees utilize mobile equipment, more contact points with customers will be created, products and services can be directly provided to customers, and services can be enhanced [104]. Frolick and Chen [105] consider that in a competitive environment, mobilization allows the organization to conduct sales and provide services to customers anytime and anywhere. Mobile equipment can provide important information to employees, so that employees working together can obtain timely information in order to make time-effective decisions. In other words, when the enterprise conducts business process reengineering (BPR): the introduction of mobile information devices enhances employees' productivity, and through mobilized knowledge sharing, BPR can become more time effective.

Based on the conclusions of this study, when an enterprise adopts mobile information devices for mobilized knowledge sharing BPR, the following advice regarding decision-making provided:

(1) Enhance perceived ease-of-use
  It can inferred from the results of regression analysis that based on the perceived ease-of-use in the technology acceptance model members of the organization will be affected by computer self-efficacy and computer playfulness. Therefore, in order to allow members of the organization to feel at ease using mobile information device, it is important to:
  (a) Enhance computer self-efficacy
    The organization could offer operational education related to and training in knowledge management systems, and promote the services of the internal IT team of the organization in order to mitigate fear of and resistance to using mobile information devices. The organization could even use a knowledge management system in order to establish and enhance the organizational members’ computer self-efficacy and their feeling of ease and willingness to use the system. This will help the organization achieve its knowledge transfer objectives.
  (b) Enhance computer playfulness
    Design of mobile information device and knowledge management system interfaces must based on intuitive and interesting principles. A user-friendly and captive design style helps the members of the organization members feel at ease when using the system. This will naturally enhance utilization.
(2) Enhance perceived usefulness
  Regarding to the aspect of system usefulness, except effects of system ease of use, this mainly comes from the perception of whether the mobile information device is substantively helpful to the employee's assigned work. Hence, the personal subjective judgment about the usefulness of a mobile information device and a knowledge management system is a key point. Therefore, the organization should strongly promote applications of mobile information devices and knowledge management systems using encouragement, and expand privileges related to using mobile information devices and knowledge management for work-related tasks, so that the use of mobile information devices and knowledge management systems could increase.
(3) System design
  Organization members' use tendencies in regard to mobile information devices and knowledge management systems are more affected by system ease of use than system usefulness. Specifically, information sharing behavior affected by personal use tendency and system usefulness. In other words, in order to enable an organization's members to participate in information sharing, organizations still must rely on both system usefulness as well as personal use tendency. When an organization's members are willing to engage in information sharing, knowledge transfer can accomplish. They will able to actively share information and learn to develop knowledge from information and thereby succeed in regard to knowledge transfer.

Generally speaking, organization can rely on the use of mobile information devices and knowledge management systems for the overall process design of work executions. They can utilize BPR so that members use self-identified common sense and their professional subjective judgement in order to utilize mobile information devices and knowledge management system at work. Matched up with interest in system interface design and education and training in information system operation and applications, the computer self-efficacy of the organization's members can be enhanced, thereby effectively affect personal technology acceptance among the organization' members. Taking this understanding of information sharing and knowledge transfer behavior into consideration, organizational knowledge can enhance with active knowledge retrieval, storage, transmission, application and innovation. Organizations can thereby further enhance their organizational knowledge competitiveness and reach sustainable operation objectives.

### Limitations and recommendations for future research

In regard to sampling representativeness, the study's selected participants worked in Taiwan. The industry sectors included educational services, finance and insurance, manufacturing, IT and communications as well as other industries. The participants' positions include all levels of the hierarchy, since enterprise sampling difficult to acquire [106]. Sampling representativeness could therefore have been more specialized. On the aspect of reliability and validity checking, some items are lower than the standards. However, in order to maintain the integrity of the design, these retained in the model. In addition, the awareness difference towered information and communication technology by gender should considered for promotion strategy [107]. Accordingly, it recommended that further studies should adopt more samples and conduct replicated reliability and validity tests in order to verify the reliability and validity of study scales. It is also recommending that future studies focus on information security and other related issues.

## Supporting information

S1 File Questionnaire–Docs. (DOCX)

## References

[1] Drucker PF. Knowledge-worker productivity: The biggest challenge. California Management Review. 1999; 41(2): 79–94.

[2] Martina K. Identification of Managerial Competencies in Knowledge-based Organizations. Journal of Competitiveness. 2012; 4(1): 129–142.

[3] Kimble C, de Vasconcelos JB, Rocha Á. Competence management in knowledge intensive organizations using consensual knowledge and ontologies. Information Systems Frontiers. 2016; 1–12.

[4] APQC. Knowledge Management: Executive Summary Consortium benchmarking study best practice report, American Productivity and Quality Center. 1999; http://www.apqc.org/knowledge-base/documents/executive-performance-management-benchmarking-report-executive-summary

[5] O'Dell C, Hubert C. The new edge in knowledge: How knowledge management is changing the way we do business. Hoboken, NJ: John Wiley & Sons; 2011.

[6] OECD. The Knowledge-Based Economy. Paris, France: OECD; 1996.

[7] Asian Productivity Organization. Training Knowledge Workers. Japan, Tokyo: Asian Productivity Organization; 2004.

[8] Snowden D. Complex acts of knowing: paradox and descriptive self-awareness. Journal of knowledge management. 2002; 6(2): 100–111.

[9] Gorelick C, Milton NJ, April K. Performance through Learning: Knowledge Management in Practice. Boston, MA: Butterworth-Heinemann; 2004.

[10] Wong KY, Aspinwall E. An Empirical Study of the Important Factors for Knowledge-Management Adoption in the SME Sector. Journal of Knowledge Management. 2005; 9(3): 64–82.

[11] Tsai SB, Xue YZ. Corporate social responsibility research among manufacturing enterprises: Taiwanese electronic material manufacturing enterprises. Applied Mechanics and Materials. 2013; 437, 1012–1016.

[12] Williams D. Models, Metaphors and Symbols for Information and Knowledge Systems. Journal of Entrepreneurship, Management and Innovation. 2014; 10(1): 79–108.

[13] Tsai SB, Lee YC., Guo JJ. Using Modified Grey Forecasting Models to Forecast the Growth Trends of Green Materials. Proceedings of the Institution of Mechanical Engineers, Part B: Journal of Engineering Manufacture. 2014; 228(6): 931–940.

[14] Tsai SB, Saito R, Lin YC, Chen Q, Zhou J. Discussing measurement criteria and competitive strategies of green suppliers from a Green Law Perspective. Proceedings of the Institution of Mechanical Engineers Proceedings of the Institution of Mechanical Engineers, Part B: Journal of Engineering Manufacture. 2015; 229(S1): 135–145.

[15] Zhou J, Wang Q, Tsai SB, Xue Y, Dong W. How to Evaluate the Job Satisfaction of Development Personnel. IEEE Transactions On Systems Man Cybernetics-Systems. 2016; doi: 10.1109/TSMC.2016.2519860

[16] Page T. Smartphone Technology, Consumer Attachment and Mass Customisation. International Journal of Green Computing. 2013; 4(2): 38–57.

[17] O’Sullivan KM. Synergy: Mobile Devices and Knowledge Management. 14.7.16 http://knowledgecompass.wordpress.com/2011/06/04/synergy-mobile-devices-and-knowledge-management/

[18] Guo JJ, Tsai SB. Discussing and Evaluating Green Supply Chain Suppliers: A Case Study of the Printed Circuit Board Industry in China. South African Journal of Industrial Engineering. 2015; 26(2): 56–67.

[19] Qu Q, Chen KY, Wei YM, Liu Y, Tsai SB, Dong W. Using Hybrid Model to Evaluate Performance of Innovation and Technology Professionals in Marine Logistics Industry. Mathematical Problems in Engineering, Article ID 361275, 8 pages. 15.9.28 http://dx.doi.org/10.1155/2015/361275.

[20] Tsai SB. Using Grey Models for Forecasting China’s Growth Trends in Renewable Energy Consumption. Clean Technologies and Environmental Policy. 2016 18, 563–571.

[21] Tsai SB, Huang CY, Wang CK, Chen Q, Pan J, Wang G, et al Using a Mixed Model to Evaluate Job Satisfaction in High-Tech Industries Plos One. 2016a; 11(5): e0154071 doi: 10.1371/journal.pone.0154071 27139697 10.1371/journal.pone.0154071 PMC4854457

[22] Davenport TH, Prusak L. Working Knowledge: How organizations Manage What They Know. Boston Massachusetts, MA: Harvard Business School Press; 1998.

[23] Du Plessis M. The strategic drivers and objectives of communities of practice as vehicles for knowledge management in small and medium enterprises. International Journal of Information Management. 2008; 28(1): 61–67.

[24] Lambe P. The unacknowledged parentage of knowledge management. Journal of Knowledge Management. 2011; 15(2): 175–197.

[25] Liebowitz J. Key ingredients to the success of an Organization’s knowledge management strategy. Knowledge and Process Management. 1999; 6(1): 37–40.

[26] Nonaka I, Peltokorpi V, Tomae H. Strategic knowledge creation: the case of Hamamatsu Photonics. International Journal of Technology Management. 2005; 30(3): 248–264.

[27] Skyrme DJ, Amidon DM. Creating the Knowledge-based Business. UK, London: Business Intelligence; 1997.

[28] Von Krogh G, Nonaka I, Aben M. Making the most of your company's knowledge: A strategic framework. Long range planning. 2001; 34(4): 421–439.

[29] Davenport TH, Klahr P. Managing customer support knowledge. California Management Review. 1998; 40(3): 195–208.

[30] Wong KY. Critical success factors for implementing knowledge management in small and medium enterprises. Industrial Management & Data Systems. 2005; 105(3): 261–279.

[31] David E, Chesebrough PE. Knowledge management: A tool for SMEs to enhance competitiveness. CACCI Journal. 2006; 1: 1–12.

[32] Edvardsson IR. Knowledge management in SMEs: The case of Icelandic firms. Knowledge Management Research & Practice. 2006; 4(4): 275–282.

[33] Paulin D, Suneson K. Knowledge Transfer, Knowledge Sharing and Knowledge Barriers—Three Blurry Terms in KM. The Electronic Journal of Knowledge Management. 2012; 10(1): 81–91.

[34] Hoof BVD, Weenen FDLV. Committed to share: commitment and CMC use as antecedents of knowledge sharing. Knowledge and Process Management. 2004; 11(1): 13–24.

[35] Chan I, Chao CK. Knowledge Management in Small and Medium Sized Enterprises. Communications of the ACM. 2008; 51(4): 83–88.

[36] Szelągowski M. Becoming a Learning Organization Through Dynamic Business Process Management. Journal of Entrepreneurship, Management and Innovation. 2014; 10(1): 147–166.

[37] Guo WF, Zhou J, Yu CL, Tsai SB, Xue Y, Chen Q, et al Evaluating the green corporate social responsibility of manufacturing corporations from a green industry law perspective. International Journal of Production Research. 2015; 53(2): 665–674.

[38] Lee YC, Hsiao YC, Peng CF, Tsai SB, Wu CH, Chen Q. Using Mahalanobis-Taguchi System, Logistic Regression and Neural Network Method to Evaluate Purchasing Audit Quality. Proceedings of the Institution of Mechanical Engineers, Part B: Journal of Engineering Manufacture. 2015; 229(S1): 3–12.

[39] Lee YC, Chu WH, Chen Q, Tsai SB, Wang J, Dong W. Integrating DEMATEL Model and Failure Mode and Effects Analysis to Determine the Priority in Solving Production Problems. Advances in Mechanical Engineering. 2016; 8(4): 1–12.

[40] Tsai SB, Xue Y, Zhang J, Chen Q, Liu Y, Zhou J, et al Models for Forecasting Growth Trends in Renewable Energy. Renewable & Sustainable Energy Reviews. 2016; doi: 10.1016/j.rser.2016.06.001

[41] David E, Chesebrough PE. Knowledge Management: A Tool for SMEs to Enhance Competitiveness. Confederation of Asia-Pacific Chambers of Commerce and Industry Journal. 2006; 1: 1–12.

[42] Maguire S, Koh SCL, Magrys A. The Adoption of e-business and Knowledge Management in SMEs. Benchmarking: An International Journal. 2007; 14: 37–58.

[43] Wu HL, Ke CC, Yung TY. The Study of Knowledge Management -A Perspective of SMEs in Taiwan. Journal for SME Development. 2009; 12: 179–122.

[44] Ministry of Economic Affairs. White Paper on Small and Medium Enterprises in Taiwan. Taipei, Taiwan: Ministry of Economic Affairs; 2015.

[45] Patnayakuni R, Rai A, Tiwana A. Systems development process improvement: a knowledge integration perspective. IEEE Transactions on Engineering Management. 2007; 54(2): 286–300.

[46] Chen WC, Chang ML, Tseng CP, Chen BC, Chang YYC. Critical human factor evaluation of knowledge sharing intention in Taiwanese enterprises. Human Factors and Ergonomics in Manufacturing & Service Industries. 2013; 23(2): 95–106.

[47] Yan Y, Davison RM, Mo C. Employee creativity formation: The roles of knowledge seeking, knowledge contributing and flow experience in Web 2.0 virtual communities. Computers in Human Behavior. 2013; 29(5): 1923–1932.

[48] Davis FD. Perceived usefulness, perceived ease of use, and user acceptance of information technology. MIS Quarterly. 1989; 13(3): 319–340.

[49] Fishbein M, Ajzen I. Belief, Attitude, Intention, and Behavior: An Introduction to Theory and Research Reading. MA: Addison-Wesley; 1975.

[50] Venkatesh V, Davis FD. A theoretical extension of the technology acceptance model four longitudinal field studies. Management science. 2000; 46(2): 186–204.

[51] Leonard-Barton D, Deschamps I. Managerial influence in the implementation of new technology. Management science. 1988; 34(10): 1252–1265.

[52] Goodhue DL. Understanding the linkage between user evaluations of systems and the underlying systems. Management Science. 1995; 41: 1827–1844.

[53] Vessey I. Cognitive Fit: A Theory-Based Analysis of the Graphs versus Tables Literature. Decision Sciences. 1991; 22(2): 219–240.

[54] Kim SH. Moderating effects of job relevance and experience on mobile wireless technology acceptance: Adoption of a smartphone by individuals. Information & Management. 2008; 45(6): 387–393.

[55] Bandura A. Social Foundations of Thought and Action: A Social Cognitive Theory. Prentice-Hall, Englewood Cliffs, NJ; 1986.

[56] Wangpipatwong S, Chutimaskul W, Papasratorn B. Understanding citizen's continuance intention to use e-government website: a composite view of technology acceptance model and computer self-efficacy. Electronic Journal of e-Government. 2008; 6(1): 55–64.

[57] Compeau DR, Higgins CA. Application of social cognitive theory to training for computer skills. Information Systems Research. 1995; 6(2): 118–143.

[58] Venkatesh V. Determinants of perceived ease of use: Integrating control, intrinsic motivation, and emotion into the technology acceptance model. Information systems research. 2000; 11(4): 342–365.

[59] Yi MY, Hwang Y. Predicting the use of web-based information systems: self-efficacy, enjoyment, learning goal orientation, and the technology acceptance model. International journal of human-computer studies. 2003; 59(4): 431–449.

[60] Atkinson M, Kydd C. Individual characteristics associated with World Wide Web use: an empirical study of playfulness and motivation. ACM SIGMIS Database. 1997; 28(2): 53–62.

[61] Eighmey J, McCord L. Adding value in the information age: Uses and gratifications of sites on the World Wide Web. Journal of Business Research. 1998; 41(3): 187–194.

[62] Henderson R, Rickwood D, Roberts P. The beta test of an electronic supermarket. Interacting with Computers. 1998; 10(4): 385–399.

[63] Teo TS, Lim VK, Lai RY. Intrinsic and extrinsic motivation in Internet usage. Omega. 1999; 27(1): 25–37.

[64] Hackbarth G, Grover V, Yi MY. Computer playfulness and anxiety: positive and negative mediators of the system experience effect on perceived ease of use. Information & management. 2003; 40(3): 221–232.

[65] Davis FD, Bagozzi RP, Warshaw PR. Extrinsic and intrinsic motivation to use computers in the workplace. Journal of Applied Social Psychology. 1992; 22(14): 1111–1132.

[66] Venkatesh V. Creation of favorable user perceptions: exploring the role of intrinsic motivation. MIS quarterly. 1999; 23(2): 218–224.

[67] Webster J, Martocchio JJ. Microcomputer playfulness: development of a measure with workplace implications. MIS quarterly. 1992; 19(2): 201–226.

[68] Igbaria M, Parasuraman S, Baroudi JJ. A motivational model of microcomputer usage. Journal of management information systems. 1996; 13(1): 127–143.

[69] Moon JW, Kim YG. Extending the TAM for a world-wide-web context. Information & Management. 2001; 38(4): 217–230.

[70] Adams DA, Nelson RR, Todd PA. Perceived usefulness, ease of use, and usage of information technology: A replication. MIS quarterly. 1992; 16(2): 227–247.

[71] Adamson I, Shine J. Extending the new technology acceptance model to measure the end user information systems satisfaction in a mandatory environment: A banks treasury. Technology Analysis and strategic management. 2003; 15(4): 441–455.

[72] Ahn T, Ryu S, Han I. The impact of the online and offline features on the user acceptance of Internet shopping malls. Electronic Commerce Research and Applications. 2005; 3(4): 405–420.

[73] Brown I, Jayakody R. B2C e-Commerce Success: a Test and validation of a revised conceptual model. Electronic Journal of Information Systems Evaluation. 2009; 11(3): 167–184.

[74] Venkatesh V, Davis FD. A model of the antecedents of perceived ease of use: Development and test. Decision sciences. 1996; 27(3): 451–481.

[75] Ajzen I, Fishbein M. Understanding attitudes and predicting social behavior. Englewood Cliffs, NJ: Prentice-Hall; 1980.

[76] Jackson CM, Chow S, Leitch RA. Toward an understanding of the behavioral intention to use an information system. Decision sciences. 1997; 28(2): 357–389.

[77] López-Nicolás C, Molina-Castillo FJ, Bouwman H. An assessment of advanced mobile services acceptance: Contributions from TAM and diffusion theory models. Information & Management. 2008; 45(6): 359–364.

[78] Hong S, Thong JY, Tam KY. Understanding continued information technology usage behavior: a comparison of three models in the context of mobile internet. Decision Support Systems. 2006; 42(3): 1819–1834.

[79] Ong CS, Lai JY, Wang YS. Factors affecting engineers’ acceptance of asynchronous e-learning systems in high-tech companies. Information & Management. 2004; 41(6): 795–804.

[80] Jung Y, Perez-Mira B, Wiley-Patton S. Consumer adoption of mobile TV: examining psychological flow and media content. Computers in Human Behavior. 2009; 25(1): 123–129.

[81] Erdelez S, Rioux K. Sharing information encountered for others on the web. The New Review of Information Behavior Research. 2000; 1: 219–233.

[82] Talja S, Hansen P. Information sharing In Spink A, & Cole C (Eds.): New Directions in Human Information Behavior (PP113-134). Dordrecht: Springer; 2006.

[83] Davis FD, Venkatesh V. Toward pre-proto type user acceptance testing of new information systems: Implications for software project management. IEEE Transactions on Engineering Management. 2004; 51(1): 31–46.

[84] Whitehill M. Knowledge-based strategy to deliver sustained competitive advantage. Long Range Planning. 1997; 30(4): 621–627.

[85] Quinn JB, Anderson P, Finkelstein S. Managing professional intellect: Making the most of the best. Harvard Business Review. 1996; 74(2): 71–80. 10156468

[86] Nonaka I, Konno N. The concept of "Ba": Building a foundation for knowledge creation. California management review. 1988; 40(3)40–54.

[87] Gilbert M, Cordey-Hayes M. Understanding the process of knowledge transfer to achieve successful technological innovation. Technovation. 1996; 16(6): 301–312.

[88] Kramer DM, Wells RP. Building networks to facilitate knowledge transfer. Science Communication. 2005; 26(4): 428–444.

[89] Hsiao RL, Tsai SDH, Lee CF. The problems of embeddedness: knowledge transfer, coordination and reuse in information systems. Organization Studies. 2006; 27(9): 1289–1317.

[90] Venkatesh V, Bala H. Technology acceptance model 3 and a research agenda on interventions. Decision Sciences. 2008; 39: 273–315.

[91] Newell A. The knowledge level. Artificial intelligence. 1982; 18(1): 87–127.

[92] Musen MA. Dimensions of knowledge sharing and reuse. Computers and biomedical research. 1992; 25(5): 435–467. 1395522 10.1016/0010-4809(92)90003-s

[93] Senge P. Sharing knowledge: the leader's role is key to a learning culture. Executive Excellence. 1997; 14: 17–17.

[94] Yu TK, Lu LC, Liu TF. Exploring factors that influence knowledge sharing behavior via weblogs. Computers in Human Behavior. 2010; 26, 32–41.

[95] Lee KC, Lee S, Kang IW. KMPI: measuring knowledge management performance. Information & Management. 2005; 42(3): 469–482.

[96] Nunnally J, Bernstein L. Psychometric Theory. New York: McGraw-Hill Higher, INC; 1994.

[97] Bland J, Altman D. Statistics notes: Cronbach's alpha. British Medical Journal. 1997; 314–275.

[98] DeVellis R. Scale development: theory and applications: theory and application. Thousand Oaks, CA: Sage; 2003.

[99] Anderson JC, Gerbing DW. Structural equation modelling in practice: A review and recommended two-step approach. Psychological Bulletin. 1988; 103(3): 411–423.

[100] Lee MKO, Cheung CMK, Chen Z. Understanding user acceptance of multimedia messaging service: An empirical study. Journal of the American Society for Information Science &Technology. 2007; 58(13): 2066–2077.

[101] Bagozzi RP, & Yi Y. On the evaluation of structural equation models. Journal of the academy of marketing science. 1988; 16(1): 74–94.

[102] Jaccard J, Wan CK. LISREL Approaches to Interaction Effects in Multiple Regression. Thousand Oaks, CA: Sage; 1996.

[103] Huang IC, Du PL, Chang CS. The Relationship between organizational climate and organizational commitment: Case studies of Kaohsiung privatized municipal hospital and three municipal hospitals. The Chinese Public Administration Review. 2003; 12(3): 27–60.

[104] Thayer G. M-commerce: Long trek to the Promised Land. Pen Computing. 2002; 9(45): 17–18.

[105] Frolick MN, Chen LD. Assessing m-commerce opportunities. Information Systems Management. 1994; 21(2): 53–61.

[106] Wu TH, Lee QC, Liu YH, Ou HM. Work values inventory instruction manual. Taipei, Taiwan: National Youth Commission, Executive Yuan, Taiwan; 1996.

[107] Verma C, Dahiya S. Gender difference towards information and communication technology awareness in Indian universities. Springer Plus. 2016; 5: 370–377. doi: 10.1186/s40064-016-2003-1 27066379 10.1186/s40064-016-2003-1 PMC4805674

