# Supplementary material for: An empirical research on relationships between subjective judgement, technology acceptance tendency and knowledge transfer
Source: PLoS One. 2017 Sep 8;12(9):e0183994. doi: 10.1371/journal.pone.0183994 (PMC5590875; doi:10.1371/journal.pone.0183994)
Supplement: S1 File — (DOCX) [file pone.0183994.s001.docx]

**Appendix**

S1: Sample Survey Items- Questionnaire

|  | Strongly Agree | Agree | Neutral | Disagree | Strongly Disagree |
| --- | --- | --- | --- | --- | --- |
|  | 5 | 4 | 3 | 2 | 1 |
| 1. **Perceived Usefulness (PU)** | | | | | |
| 1.使用智慧型手機或平板電腦能改進我的工作成果。  My work can be improved by using a smart phone or a tablet computer. | [ ] | [ ] | [ ] | [ ] | [ ] |
| 2.使用智慧型手機或平板電腦能提高我的工作生產力。  My productivity can be improved by using a smart phone or a tablet computer. | [ ] | [ ] | [ ] | [ ] | [ ] |
| 1. **Perceived Ease-of-Use (PEOU)** | | | | | |
| 1.我覺得智慧型手機或平板電腦的功能是簡單易懂的。  I feel that the functions of the smart phone and the tablet computer were easy to understand. | [ ] | [ ] | [ ] | [ ] | [ ] |
| 2.運用智慧型手機或平板電腦進行分享(資料)是很容易的。  It is easy to share information by using a smart phone or a tablet computer. | [ ] | [ ] | [ ] | [ ] | [ ] |
| 1. **Subjective Judgment (SJ)** | | | | | |
| 1.公司具有影響力的主管支持我運用智慧型手機或平板電腦轉發公司重要文件或資訊。  My forwarding of official documents and/or information via smart phone or tablet computer was supported by the influential managers of my company. | [ ] | [ ] | [ ] | [ ] | [ ] |
| 2. 公司同仁建議我嘗試利用智慧型手機或平板電腦來提高工作效率。  My colleagues have suggested to me that I can improve my efficiency by using a smart phone or a tablet computer. | [ ] | [ ] | [ ] | [ ] | [ ] |
| 1. **Computer Self-Efficacy (CSE)** | | | | | |
| 1.既使周遭沒有其他人告訴我使用智慧型手機或平板電腦的方法，我仍然會克服。  Even though nobody can teach me how to use the smart phone or the tablet computer , I will proceed. | [ ] | [ ] | [ ] | [ ] | [ ] |
| 2.透過智慧型手機或平板電腦的說明書，我就會使用了。  I can operate the smart phone or the tablet computer by following the instruction manual. | [ ] | [ ] | [ ] | [ ] | [ ] |
| 1. **Computer Playfulness (CPLAY)** | | | | | |
| 1.我覺得使用智慧型手機或平板電腦非常得心應手。  I can handle a smart phone or a tablet computer very well. | [ ] | [ ] | [ ] | [ ] | [ ] |
| 2.我覺得使用智慧型手機或平板電腦可以激發個人創意。  I think that my creativity is positively affected by using a smart phone or a tablet computer. | [ ] | [ ] | [ ] | [ ] | [ ] |
| 1. **Usage Intention (UI)** | | | | | |
| 1.我偏愛能執行最新應用程式的智慧型手機或平板電腦。  I prefer to use s smart phone or tablet computer that can run advanced applications (Apps). | [ ] | [ ] | [ ] | [ ] | [ ] |
| 2.我期待擁有能執行最新應用程式的智慧型手機或平板電腦。  I expect to use a smart phone or tablet computer that can run advanced Apps. | [ ] | [ ] | [ ] | [ ] | [ ] |
| 1. **Information Sharing Behavior (ISB)** | | | | | |
| 1.我曾經提供知識、訊息或資訊給工作團隊成員。  I share my knowledge, share messages, or share information with colleagues. | [ ] | [ ] | [ ] | [ ] | [ ] |
| 2.我經常主動地與其他組織成員分享知識、訊息或資訊。  I often share knowledge, messages or information with other members of the organization. | [ ] | [ ] | [ ] | [ ] | [ ] |
| 1. **Knowledge Transfer (KT)** | | | | | |
| 1.企業主張透過智慧型手機或平板電腦分享資訊與知識以滿足工作任務需求。  Company representatives are advocating that we satisfy task demand by using a smart phone or a tablet computer. | [ ] | [ ] | [ ] | [ ] | [ ] |
| 2.企業主張透過智慧型手機或平板電腦分享資訊與知識以提升工作效率。  Company representatives are advocating that we work efficiently by using smart phone or tablet computer. | [ ] | [ ] | [ ] | [ ] | [ ] |
